# Neurally Encoding Time for Olfactory Navigation

**DOI:** 10.1371/journal.pcbi.1004682

**Published:** 2016-01-05

**Authors:** In Jun Park, Andrew M. Hein, Yuriy V. Bobkov, Matthew A. Reidenbach, Barry W. Ache, Jose C. Principe

**Affiliations:** 1 Department of Electrical and Computer Engineering, University of Florida, Gainesville, Florida, United States of America; 2 Department of Ecology and Evolutionary Biology, Princeton University, Princeton, New Jersey, United States of America; 3 Whitney Laboratory for Marine Bioscience, University of Florida, St. Augustine, Florida, United States of America; 4 Center for Smell and Taste, and McKnight Brain Institute, University of Florida, Gainesville, Florida, United States of America; 5 Department of Environmental Sciences, University of Virginia, Charlottesville, Virginia, United States of America; 6 Departments of Biology and Neuroscience, University of Florida, Gainesville, Florida, United States of America; Northeastern University, UNITED STATES

## Abstract

Accurately encoding time is one of the fundamental challenges faced by the nervous system in mediating behavior. We recently reported that some animals have a specialized population of rhythmically active neurons in their olfactory organs with the potential to peripherally encode temporal information about odor encounters. If these neurons do indeed encode the timing of odor arrivals, it should be possible to demonstrate that this capacity has some functional significance. Here we show how this sensory input can profoundly influence an animal’s ability to locate the source of odor cues in realistic turbulent environments—a common task faced by species that rely on olfactory cues for navigation. Using detailed data from a turbulent plume created in the laboratory, we reconstruct the spatiotemporal behavior of a real odor field. We use recurrence theory to show that information about position relative to the source of the odor plume is embedded in the timing between odor pulses. Then, using a parameterized computational model, we show how an animal can use populations of rhythmically active neurons to capture and encode this temporal information in real time, and use it to efficiently navigate to an odor source. Our results demonstrate that the capacity to accurately encode temporal information about sensory cues may be crucial for efficient olfactory navigation. More generally, our results suggest a mechanism for extracting and encoding temporal information from the sensory environment that could have broad utility for neural information processing.

## Author Summary

Many animals navigate turbulent environments using odor cues, a behavior known as olfactory search. We propose a neural mechanism for olfactory search based on evidence that a functional subset of olfactory receptor neurons (ORNs) called bursting ORNs or bORNs can encode the time intervals between successive encounters with odor. We show that these time intervals are estimators of the recurrence time, an information-rich statistic of the turbulent flow. Using a computational model parameterized with data from an actual turbulent plume, we demonstrate that a searcher can locate an odor source efficiently using only input from bORNs. These findings provide scientific evidence that the most important navigational information captured by the olfactory system may come in the form of measurements of time.

## Introduction

There are four fundamental dimensions to all sensory modalities—quality, quantity, space and time. While the quality and quantity dimensions of olfaction are well appreciated and increasingly understood, it has long been assumed that olfaction yields little information about space and time. In contrast, in vision and audition, neural encoding of space and time information provides effective perception of the dynamic world [1, 2], commonly referred to as ‘scene analysis’ [3]. Since olfaction presumably is the oldest sensory system (e.g., [4]), it would be surprising if animals relying heavily on olfaction did not evolve some version of ‘olfactory scene analysis’ as an edge for survival [5]. Indeed, many animals, including humans (e.g. [6]) are capable of using odor cues to navigate. The best-studied example of this type of navigation is known as olfactory search, a behavior in which animals locate the source of an odor emitted by food or potential mates (e.g., [7–11]). For all but the smallest animals, searches take place in turbulent air or water. The considerable difficulties associated with finding an odor source in turbulence have been well documented (see e.g., [12–14]). The question is, how do organizational features inherent in the olfactory system allow animals to accomplish this task?

Past studies of olfactory search have generally either proposed navigational algorithms and demonstrated their efficiency in idealized environments (e.g., [12, 13, 15]), or studied behavioral responses to controlled scent stimuli (e.g., [10, 16, 17]). These studies have yielded general principles of search and greater knowledge of the behavioral responses of searchers to odor cues. Yet, it is still not clear what features of odor cues animals actually measure, neurally encode, and use for navigation. Here, we suggest that reverse-engineering search strategies from the neurophysiology of the olfactory system may provide a way forward.

In vision, the relative motion of objects provides information about the spatial structure of the environment and animals use this information to navigate. The head related transfer function serves a similar purpose in audition. In the case of olfaction, the time intervals between odor encounters inherent in the structure of odor plumes (i.e., odor intermittency) can vary dramatically with distance to odor sources and therefore appear to be candidate cues for olfactory navigation (e.g., [14, 18]). If this is generally the case, one could hypothesize the existence of a specialized sensory subsystem that could capture and represent timing of past odor encounters. We recently demonstrated [19] that a subset of olfactory receptor neurons (ORNs)—known as ‘bursting’ ORNs (bORNs) because they spontaneously and rhythmically oscillate and are entrained by odorants—have the capacity to encode time intervals between odor encounters. bORNs have been identified in a diverse range of animals including arthropods [20], amphibians [21], and mammals [21–24], suggesting that they may provide an important and basic function in the olfactory system. The finding that bORNs appear to be capable of capturing information about the timing of odor encounters supports the hypothesis that animals have evolved a functionally distinct sensory subsystem with the capacity to accurately measure and encode the times between odor arrivals. However, whether this capability is related to the navigational challenges that animals face in natural odor environments, and precisely how it could influence search behavior is an open question. We address this question in what follows.

**Fig 1:**
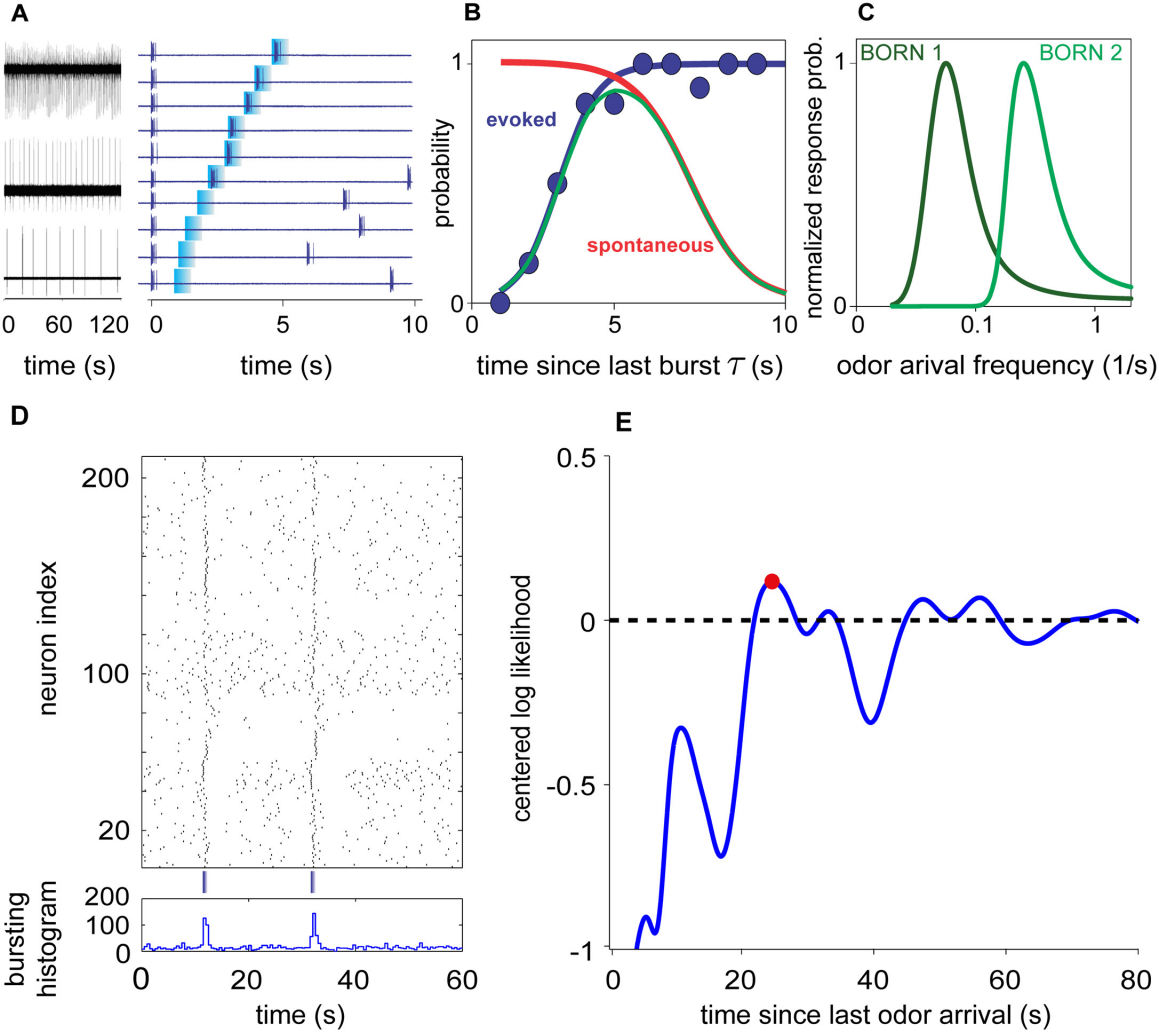
Encoding and decoding time since the last odor encounter from a population of bORNs (experimental data from the spiny lobster *Panulirus argus*). (A) Electrophysiological recordings of spontaneous bursting from three bORNs with different intrinsic burst frequencies (left), and bursting pattern of a single bORN (right) stimulated with odor (blue marks). Trials aligned in order of increasing time since last burst (bottom to top). Note that bORN does not respond to stimulus when time since last burst is short (bottom 4 trials) and instead, continues to burst spontaneously, (B) Probability of bursting in response to odorant as a function of time since last burst *τ*. Blue points are electrophysiologial data; blue line is sigmoid fit to data. Red curve represents the probability that the bORN will go *τ* seconds before bursting spontaneously (1—CDF of spontaneous inter-burst interval). Together, these curves tune the bORN to be most sensitive to odors that arrive with a particular frequency. (C) Probability of bursting in response to a stimulus as a function of stimulus frequency for two bORNs tuned by different evoked and spontaneous burst functions. (D) Raster plot (upper) and burst histogram (lower) of a heterogeneous population of 210 bORNs constructed from multiple single-neuron electrophysiological recordings showing spontaneous bursting and responses to odor stimuli (blue marks). This reconstructed population of bORNs encodes time between two odor stimuli (20.7 s). (E) The time interval between odor stimuli can be decoded from the bursting pattern of a heterogeneous bORN population shown in (D) using a simple maximum likelihood procedure (decoded interval is 23.2 s). Data are from [19].

Bursting olfactory receptor neurons exhibit several functional properties that suggest they may serve to measure and encode the timing of odor cues. Unlike canonical, tonic ORNs whose activity follows the concentration of a stimulus or the rate of change in concentration [25], bORNs burst spontaneously, even in the absence of odor stimulation, in addition to bursting in response to odors. Each bORN’s spontaneous activity is characterized by a distinct intrinsic bursting frequency (Fig 1A), and whether a bORN responds to an odor stimulus depends on when the odorant arrives relative to its inherent bursting cycle (Fig 1A). The probability that a bORN will burst in response to an odor increases strongly as a function of the time since its last burst *τ* (Fig 1A and 1B). The bORN’s probability of responding to a stimulus can be characterized by two functions: the evoked response probability (Fig 1B, blue curve and points) and the probability of going *τ* seconds without bursting spontaneously (Fig 1B, red curve). The composition of these two functions gives rise to a ‘time entrainment tuning curve’ for that bORN (Fig 1B, green curve). The population of bORNs is heterogeneous, creating an ensemble sensitivity to a wide range of odor arrival periodicities that can extend from hundreds of milliseconds to tens of seconds (Fig 1C). As a population, bORNs encode in their pattern of bursting the time since the last odor was encountered (Fig 1D). This neurally encoded time interval can be decoded using a simple maximum likelihood procedure, implemented, for example, with a winner take all operation on the population of neurons that receives axonal projections from the bORNs (Fig 1E). Unlike other proposed methods for neurally encoding time intervals, which require precise fine-tuning of ensembles of neurons (e.g., [26, 27]), bORN-based encoding requires no such fine-tuning and yields low variance in the estimate of time, even for long time-intervals between odor encounters [19]. This means that the time since the last odor encounter can be measured, encoded, and decoded with high accuracy, even when odors arrive infrequently. bORNs represent a sensory-specific timing mechanism [28] that provide animals that have them [21–24, 29] with the ability to peripherally encode the time intervals between odor encounters. The central hypothesis we test in this manuscript is whether bORNs provide a neural mechanism for extracting useful navigational information in natural turbulent odor environments.

Here we combine a model based on neurophysiological measurements obtained from the spiny lobster *Panulirus argus* [19] with detailed data from a real turbulent plume to show that populations of bORNs can directly measure properties of odor intermittency that are useful for navigation. We use detailed data on the concentration of a fluorescein dye from planar laser-induced fluorescence (PLIF) recordings from a turbulent plume [18] to rigorously characterize the timing of odor arrivals. Our analysis shows that, at scales relevant to animals searching for odor sources, there is sufficient information in the timing of odor arrivals to distinguish different locations in the plume. Finally, we use a computational model parameterized with experimental measurements from *P. argus* bORNs and the turbulent plume data to show that a searching animal with paired olfactory organs can quickly locate an odor source using the real-time measurements of odor intermittency captured by bORNs.

## Results

### Extracting navigational information from a turbulent odor field

**Fig 2:**
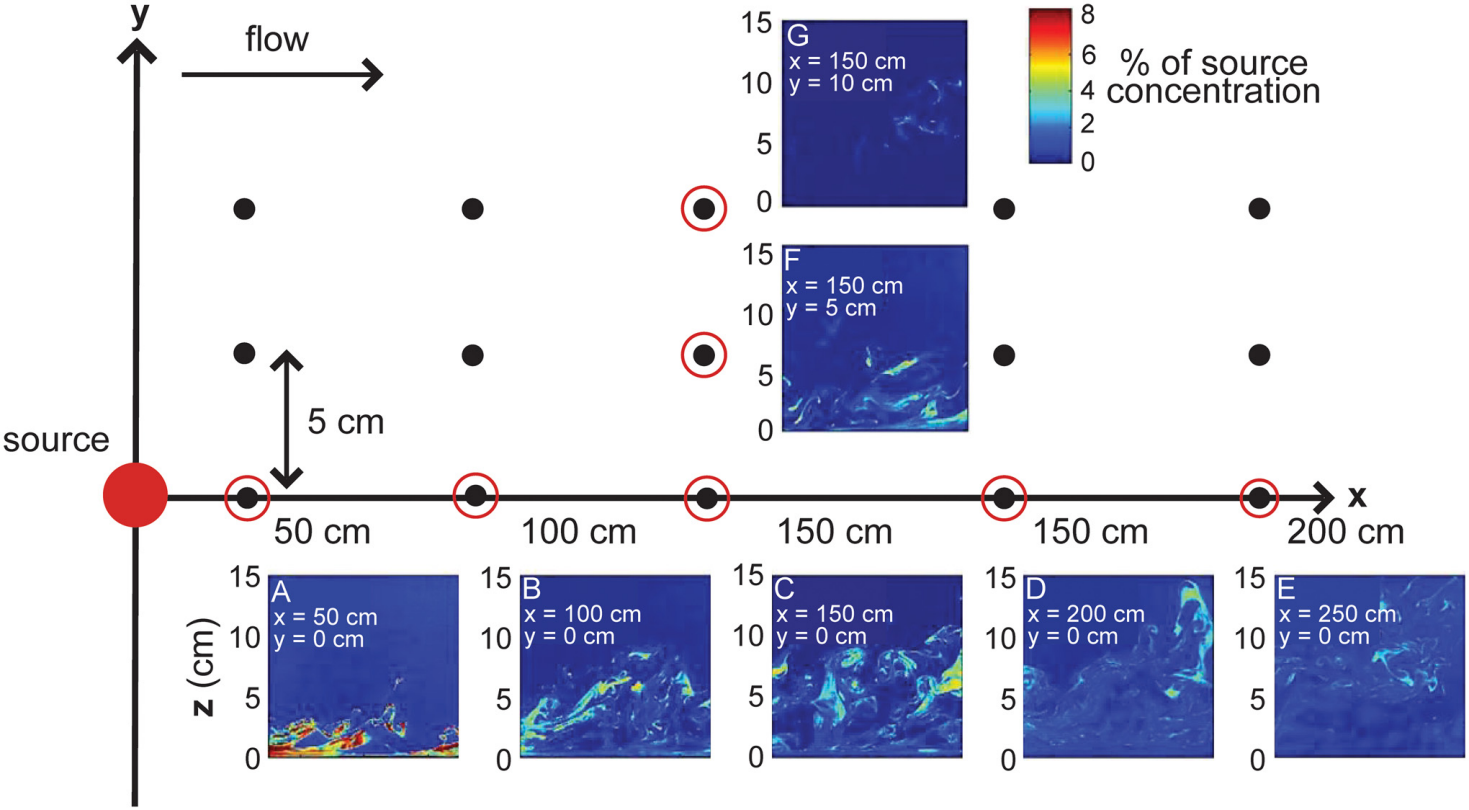
Odor plume PLIF videos taken at 15 locations. Instantaneous odor concentration (expressed as % of source concentration) at (A) x = 50 cm, (B) x = 100 cm, (C) x = 150 cm, (D) x = 200 cm, (E) x = 250 cm from the source along the odor plume centerline, and (F) y = 5 cm, (G) y = 10 cm from the odor plume centerline at x = 150 cm.

Turbulent odor plumes in nature have a large range of odor frequencies; time periods between the arrival of bursts of high odor concentration can exist from milliseconds to many seconds [30, 31]. An animal traveling in such an environment could potentially measure many different features of the odor landscape. To determine whether bORNs are capable of measuring particular features of the odor field that contain useful navigational information, we use PLIF (planar laser-induced fluorescence) videos recorded at 15 different sites in a large laboratory flume (dimensions: 25 m long, 0.6 m wide, and 0.3 m deep) into which fluorescein dye was released to mimic an odorant (see [18] and Materials and Methods for details). The flow conditions and plume created by dye release were chosen to mimic plumes experienced by lobsters under natural foraging conditions [18]. From pixel intensities in the movies, we extracted a time series of fluorescence intensity at each of the 15 sites (Fig 2) and used these time series to characterize the dynamic behavior of dye in the turbulent plume (see Materials and Methods). We assume that the intensity of fluorescence is equivalent to odor concentration and we use these terms interchangeably.

**Fig 3:**
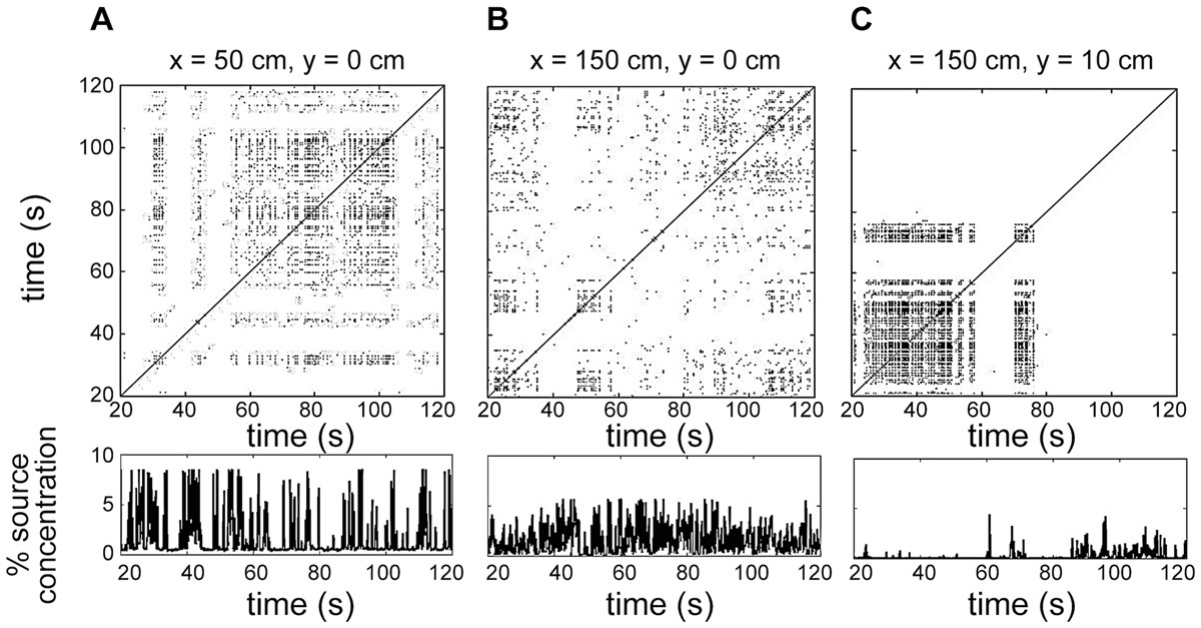
Recurrence plots (upper panels) and corresponding concentration time series (lower panels) for selected locations in the plume: (A) x = 50 cm, (B) x = 150 cm from the source along the plume centerline, and (C) y = 10 cm from the centerline at x = 150 cm, where height is 2.5 cm from the substratum. Black points in the recurrence plots indicate that recurrence occurs; white regions indicate that recurrence does not occur (see Results).

Unlike steady concentration gradients, turbulent odor plumes are characterized by large fluctuations in odor concentration at any point in space. A biological or artificial sensor suspended in the plume will register a time series of odor measurements characterized by bursts, in which the odor concentration well exceeds its mean value, and “blanks,” in which concentration is very low relative to its mean (Fig 3 lower panels, [14]). These large fluctuations in concentration mean that the organism must measure concentration for a long period of time in order to accurately estimate mean odor concentration far from the odor source (see e.g., [13]). An alternative to measuring odor concentration itself is to measure the time intervals during which odor concentration is below detectable threshold [13, 15, 32]. If the arrivals of detectable odor bursts were periodic, the periodicity of odor arrival (e.g., the inter-arrival period) would be a natural metric for measuring the time intervals. In turbulent flows, however, the arrival of bursts is not perfectly periodic. Instead, we employ a concept from dynamical systems theory known as recurrence, which extends the concept of periodicity to events that reoccur in time but do not necessarily follow a regular periodic cycle. The recurrence time provides a generalization of the inter-event period (the period between arrivals of whiffs of odor above a detectable threshold in this case), and, as we will show below, recurrence time turns out to be a statistical property of the odor field that can be estimated and encoded peripherally by bORNs.

Measuring recurrence time of the odor plume data requires that we be able to characterize the time between “similar” events in the concentration time series. This, in turn, requires that we define what it means for two time periods in an odor time series to be similar. Dynamical systems theory provides a rigorous method for doing this. In particular, we define a criterion for determining whether two points in the odor time series are similar enough to be considered recurrences by characterizing a dynamical invariant of the turbulent flow known as the attractor (see e.g., [33, 34] for a detailed discussion). Any point in the odor time series can be mapped to a corresponding point on the attractor, which is a high-dimensional object that characterizes the dynamical behavior of concentration over time. Two points that are close to one another on the attractor are considered to be similar if they fall within a specified threshold distance of one another. The time required for the time series to revisit such similar points is the recurrence time. To reconstruct the attractor from the time series of odor concentration measurements at each position in space we use Takens’ delay embedding theorem [35], which creates a bijective mapping between the time series and a sufficiently high-dimensional attractor (10 dimensions in this case; details of attractor reconstruction are described in S1 Appendix, [33]). After reconstructing the attractor, we can define formally what it means for two points in time to be similar using recurrence theory ([36, 37] and extension by Eckmann *et al*. [38]). The recurrence plot is a matrix **R** that quantifies the dynamics of the turbulent flow and can be measured locally by

$$R\left(i,j\right)=\Theta (r-||x_{i}-x_{j}||),\;i,\;j=1,2,...N,$$ (1)

where *r* is an allowable neighborhood distance, Θ is a Heaviside function, and ||**x**_*i*_ − **x**_*j*_|| denotes a Euclidean distance between **x**_*i*_ and its translated version across time **x**_*j*_. The Heaviside function provides a value of one (**R**(*i*, *j*) = 1) when the difference between **x**_*i*_ and **x**_*j*_ is smaller than *r*, and zero for all other cases. To estimate recurrence of trajectories at a given concentration, we open a similarity sphere of radius *r*, around a reference value **x**_*i*_. When the concentration falls within this sphere at a later time, a recurrence occurs (black points in Fig 3, see also S1 Appendix).

The recurrence plot provides a visual representation of the self-similarity of the odor arrival time series (instantaneous odor concentrations at different spatial locations are shown in Fig 3; upper panels are recurrence plots, lower panels are the corresponding concentration time series). The essential observation is that the features of the odor plume shown in Fig 3 vary dramatically, both with distance to the source (compare Fig 3A and 3B) and distance from the plume centerline (compare Fig 3B and 3C). This implies that the timing of odor arrivals contains structure that could, in principle, be used to determine position relative to the odor source. This empirical result, obtained using methods from dynamical systems theory, is consistent with theoretical results from statistical fluid dynamics [14].

**Fig 4:**
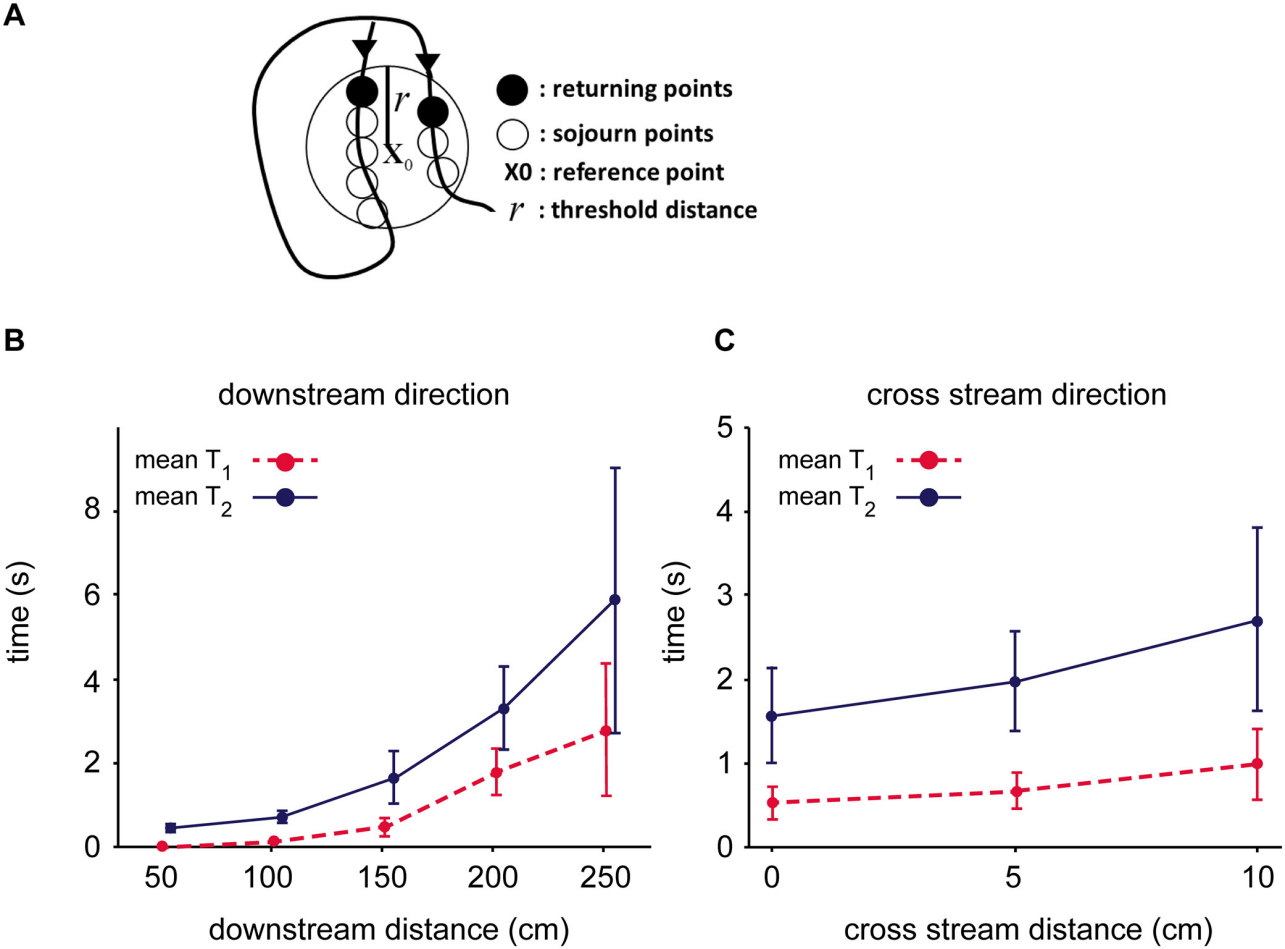
Recurrence time and position in the plume. (A) Example trajectory in reconstructed phase space. Two types of recurrence time index are obtained by averaging time intervals between all successive recurrent points (${\bar{T}}_{1}$) or only returning points (${\bar{T}}_{2}$) in a circle of radius *r* centered at a reference point **x**_0_. To estimate ${\bar{T}}_{1}$, the refractory period of at least some bORNs in the population would need to be short relative to the times between successive recurrence points. ${\bar{T}}_{2}$ can be estimated with longer refractory periods. (B) Mean (points) and standard deviation of ${\bar{T}}_{1}$ and ${\bar{T}}_{2}$ indices for positions in downstream direction. (C) Mean and standard deviation of ${\bar{T}}_{1}$ and ${\bar{T}}_{2}$ indices for positions in cross-stream direction.

Given the differences in recurrence behavior in different regions of the plume (Fig 3), an immediate question is whether the sensory capabilities of bORNs could allow an animal to measure features of this structure that are useful for navigation. A searcher moving through a plume must decide, in real time and with local measurements, whether it is traveling in the right direction and adjust its movements accordingly [12]. Rather than recording a stationary time series of odor encounters, a moving animal will experience a sequence of encounters that is time-varying (i.e., the rate of odor arrivals changes as the animal moves from one location in the plume to another). Recurrence theory suggests a solution to this problem: recurrence time—the time needed for a trajectory to revisit the same area in phase space [39, 40]—is a sensitive metric for quantifying the degree to which the dynamics of a time series change over time. We consider two types of recurrence time statistic that are consistent with the known functional properties of bORNs: the mean recurrence times of first and second types, which we will denote ${\bar{T}}_{1}$ and ${\bar{T}}_{2}$ [39]. From a specific trajectory in the reconstructed state space produced through time-delay embedding, we select a reference point **x**_0_. Points that fall within the region defined by {**x**: ||**x** − **x**_0_|| < *r*} are deemed similar to the reference point (the points within a distance *r* of the reference point shown in Fig 4A). These points define a set of trajectories **S**_**1**_ = {**x**_*t*_1__, **x**_*t*_2__, …, **x**_*t*_*i*__, …}. The recurrence time of the first type is simply computed by subtracting successive times in the subset: {*T*_1_(*i*) = *t*_*i*+1_ − *t*_*i*_, *i* = 1, 2, …}. ${\bar{T}}_{1}$ is the average of these return time intervals. By removing from the count the successive points inside the neighborhood, called sojourn points, we obtain a new set $S_{2}=\left\{x_{t_{1}^{\prime }},x_{t_{2}^{\prime }},...,x_{t_{i}^{\prime }},...\right\}$ that is composed of only returning points (black-filled circle in Fig 4A). The recurrence times of the second type ${\bar{T}}_{2}$ can be computed by averaging intervals between return times of {*T*_2_(*i*) = *t*_*i*′+1_ − *t*_*i*′_, *i* = 1, 2, …}. Heuristically, ${\bar{T}}_{1}$ is the average time taken for the odor concentration time series experienced by the searcher to revisit a similar point in phase space. To measure ${\bar{T}}_{1}$ exactly, a population of bORNs would need to be able to resolve the time intervals between all similar points in the odor time series, even if these points occur in short succession. By contrast, ${\bar{T}}_{2}$ excludes points that occur in short succession (Fig 4A, sojourn points are excluded), as one might expect if bORNs burst in response to an odor detection, but remain refractory if the next odor whiff arrives shortly thereafter. Because the precise refractory characteristics of entire populations of bORNs are not fully characterized, we include both of these metrics. To investigate whether ${\bar{T}}_{1}$ and ${\bar{T}}_{2}$ contain navigational information using the flume data, we assumed the detection threshold corresponded to a dye concentration of 2.55% of source concentration and *r* = 0.33% (S1 Appendix). It is not possible to relate this value directly to the bORN odor sensitivity threshold because dye concentration serves only as a surrogate for odor concentration (see Materials and Methods). Fig 4B and 4C show the mean and standard deviation of ${\bar{T}}_{1}$ and ${\bar{T}}_{2}$ for downstream and cross-stream positions. The mean of ${\bar{T}}_{1}$ and ${\bar{T}}_{2}$ increases with increasing distance from the source or plume centerline, which indicates the recurrence time contains information about where the animal is located relative to the odor source.

Fig 4 illustrates that there is navigational information inherent in ${\bar{T}}_{1}$ and ${\bar{T}}_{2}$ when these metrics are computed from an embedded version of the odor time series. We use embedding because it ensures that the full information contained within the odor time series is preserved; yet, it is unclear whether an organism such as *P. argus* could perform the neural computation required to generate such an embedding. However, many recurrence metrics used to identify changes in the dynamics of time series exhibit an interesting property: these metrics can generally be reliably estimated directly from the original time series without embedding [34]. This means that the time intervals encoded directly by bORNs may serve as effective estimators of the recurrence time. In particular, a subset of bORNs in the population will burst in response to an odor concentration that exceeds a threshold, which serves as the reference concentration **x**_0_, selecting implicitly a trajectory of constant concentration in the turbulent flow where the animal is located. As described above, the next time that the bORNs burst in response to odor concentration **x**_0_, the population encodes the time since the last odor encounter, which can be decoded by maximum likelihood. This time corresponds to a stochastic estimation of mean recurrence time (${\bar{T}}_{1}$ or ${\bar{T}}_{2}$) of a trajectory in the flow at the particular odor concentration that triggered the bORN and will be denoted ${\hat{T}}_{1}$ and ${\hat{T}}_{2}$ respectively. If bORNs were able to respond instantaneously to odor arrivals, regardless of the time at which the last odor arrived, the bORN population would estimate ${\hat{T}}_{1}$; however, because cells have a minimum neural refractory period, the population filters out up-crossings that occur in short succession, making the estimate closer to ${\hat{T}}_{2}$.

### Using bORNs to find an odor source

**Fig 5:**
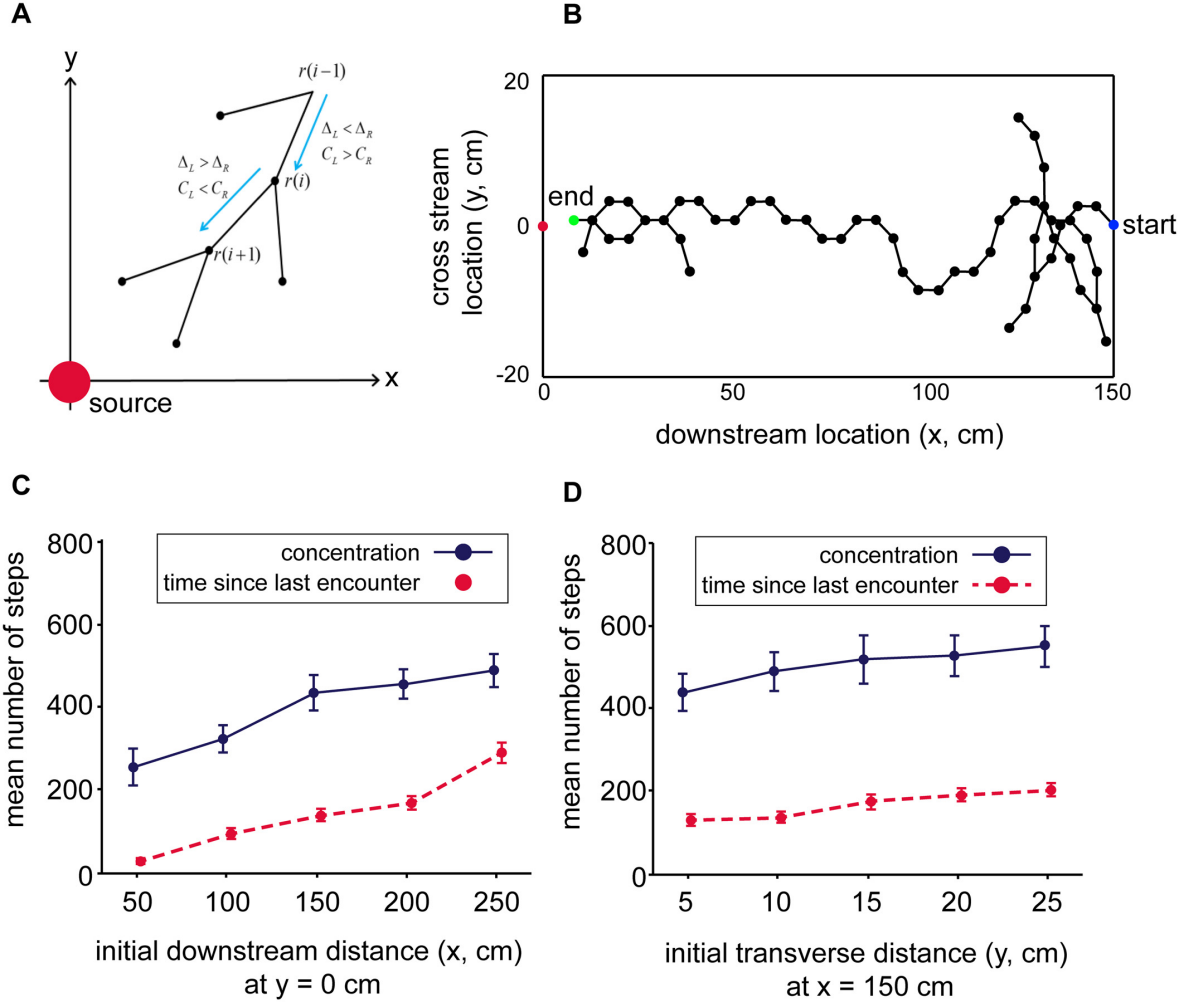
Search strategy using bORNs or measurements of concentration. (A) Search strategy based on a pair of sensors. The searcher compares measurements of the time since the last odor arrival, Δ, or measurements of concentration, *C*, registered by left and right sensors and steers in the direction of the shorter time or higher concentration. (B) Example trajectory of bORN-based strategy (initial position is x = 150 cm, y = 0 cm). The searcher begins at the “start” point and stops at the “end” point. The trajectory is continuous, with decisions made at every dot. Points that end outside the plume indicate that the searcher backtracks to its previous position. (C, D) Mean and standard error for number of steps required for strategies based on measurements of concentration (blue) and time since last encounter (bORN strategy, red) as a function of starting location relative to the source.

To determine whether an animal could use the recurrence times estimated by bORNs to navigate, we use a computational search model parameterized with data from *P. argus* neurophysiology and a simulated environment based on the turbulent plume data (see Materials and Methods). At each time step, the searcher determines its movement direction by comparing bilateral measurements of the scent field (Fig 5A; [17]). We study a strategy with two sensors because lobsters have been shown to exhibit longer search times and far more tortuous search paths when one of their olfactory organs is ablated, suggesting that bilateral comparisons of odor measurements (i.e., tropotaxis) is an important component of their search behavior [41]. The searcher probes the odor plume using its two sensors and waits a maximum observation time for an odor encounter; otherwise it returns to its previous position to avoid leaving the plume (see Materials and Methods; similar behavior, in which lobsters that exit a plume turn to re-enter it has been observed experimentally [41]). We consider two quantities that a searcher could measure: time since the last odor encounter, which can be measured by bORNs, and concentration, which can be measured by ordinary olfactory receptor neurons. Strategies based on scent concentration are discussed extensively in the literature (e.g. [42]) and we include such a strategy for reference. In the strategy based on the time since the last encounter, the searcher steers in the direction of the sensor that measures smaller recurrence time, which the searcher estimates locally by the time since the last encounter measured by each sensor. In the strategy using instantaneous concentration, the searcher moves to the direction of the sensor measuring the larger instantaneous concentration (Table B in S1 Appendix). For comparison, we also study the performance of single sensor strategies (S1 Appendix).

Fig 5B shows an example trajectory of a searcher that uses time since last encounter. The casting pattern in the trajectory (i.e., zigzagging across the plume) resembles trajectories of real olfactory searchers (e.g., [43, 44]). For both strategies, the number of steps increases as the downstream distance from the source increases (Fig 5C). However, the strategy based on time since last encounter requires far fewer steps to locate the source. Strategies based on a single olfactory sensor are still capable of finding the odor source, but take far longer (S1 Appendix), which is consistent with experiments showing that lobsters with only one functional antenna take longer to reach a scent source [41]. Notably, the strategy that relies on time since last encounter depends only very weakly on the distance to the plume center-line (Fig 5C), an important feature given that there is no guarantee that an odor source will be directly up current. Although olfactory searchers likely implement search strategies that are more complex than the simple strategy explored here (e.g., [45]), Fig 5 demonstrates that the statistic measured by bORNs is sufficient to lead a searcher quickly to a scent source, even in the absence of any other measurements of the search environment (e.g., flow direction).

## Discussion

Our results suggest that bursting olfactory receptor neurons serve a crucial but previously unappreciated role in olfactory navigation by accurately encoding the time intervals between odor encounters. In real turbulent odor plumes like the one studied here, there is structure in the sequence of odor arrivals at any given location (e.g., Fig 3; [14, 18]) and this structure is strongly correlated with position relative to the odor source. Navigational information contained in this structure can be captured by a simple metric: recurrence time (the $\bar{T_{1}}$ and $\bar{T_{2}}$ metrics discussed above, Fig 4). bORNs are capable of collectively encoding the time since the last odor encounter (Fig 1) and this quantity is precisely a low-dimensional stochastic estimate of recurrence time. The implication is that the lobster has evolved a specialized sensory subsystem that is highly sensitive to changes in the local structure of turbulent odor plumes [39, 40].

A searcher employing a simple heuristic that uses only the information captured by bORNs can quickly and reliably locate an odor source in a realistic turbulent plume (Fig 5). This demonstrates that the interval between odor encounters is useful for solving the online navigational problem animals actually face when searching a turbulent environment—to locate the source without wasting time waiting in places where the likelihood of encountering an odor is low [12, 15]. Recurrence times are ideally suited to this task because, unlike other metrics that are typically applied to time series analysis, recurrence times are highly sensitive to changes in the rate of arrival of odor pulses (i.e., nonstationarity of the attractor, [39]), which occur when the searcher actively moves through the plume as it samples odors. Though a searcher could also use measurements of odor concentration, strategies based on concentration estimates alone perform poorly (Fig 5, Fig C in S1 Appendix) suggesting, counterintuitively, that the most important navigational information captured by the olfactory system may come in the form of measurement of time rather than measurement of concentration.

It is likely that our findings apply to other species of olfactory searchers in different types of turbulent odor environments (e.g., air versus water). Using a very different approach from that taken here, Celani *et al*. [14] applied methods from statistical fluid dynamics to characterize the features of odor transport in idealized turbulent plumes that are believed to be most relevant for olfactory navigation. While the mean odor concentration and the probability distribution of concentration varies systematically with position relative to an odor source, the manner in which these statistical features of the plume change with distance to the source are strongly influenced by properties of the plume such as the mean speed of advection (i.e. flow rate) and the amount of odorant released at the source. By contrast, the duration of time intervals during which the odor is below a detection threshold (analogous to the ${\hat{T}}_{2}$ statistic defined above) depends far less strongly on the details of the environment, which implies that there is information embedded in this statistic that can be extracted without knowledge of the properties of the flow and odor source. A second advantage of navigating using odor inter-arrival times is that these intervals decay rapidly with distance from the plume mid line (e.g. Fig 3), whereas other features of the odor field do not [14]. The statistical fluid dynamics approach is complementary to our method of empirically characterizing the dynamics of odor concentration using dynamical systems theory. Moreover, the results of [14] suggest that our general conclusion—that odor inter-arrival times are a sensitive metric for navigating odor plumes, and therefore, that bORNs can encode useful navigational information—is likely to extend to environments that differ substantially from the laboratory plume studied here (e.g. different flow speeds, odor concentrations, air vs. water, differences in chemical diffusivity).

Animals that engage in olfactory search also use information from other sensory modalities to guide search behavior. For example, male moths locate females using measurements of prevailing winds in addition to measurements of the pheromones females emit [14] and mosquitos combine visual and thermal cues with CO_2_ detection to localize hosts [46]. In the case of most species that navigate using olfactory cues, it remains to be shown precisely how information from multiple sensory modalities is integrated to govern movement decisions. Various strategies for olfactory navigation have been proposed (e.g., the “mapless” scheme of [45], the “infotaxis” scheme of [13], the signal-modulated random walks studied in [15, 32]), but at present, the behavioral and neurophysiological data necessary to evaluate such navigational strategies and compare them to one another is lacking. However, the utility of odor inter-arrival times and the existence of a sensory subsystem capable of measuring them directly strongly suggest that temporal information inherent in the olfactory signal itself is fundamental to the search process. The presence of bORNs in animals as phylogenetically diverse as arthropods [20], amphibians [21], and mammals [21–24] suggests that the dynamic encoding of temporal information these neurons provide may even be fundamental to olfactory navigation.

Taken together, our results reveal a neural mechanism for extracting and encoding navigational information from a noisy sequence of odor encounters. They add to an increasing understanding of how complex olfactory data are captured, encoded, and relayed through the brain [17, 47, 48]. Our results argue strongly that the ability of bORNs to encode time not only has behavioral significance, but that the dimension of time, and through time, the dimension of space, is inherent in olfaction. Thus, olfactory scene analysis is not limited to the sensory dimensions of quality and quantity (e.g., [49]) but also appears to employ the spatial and temporal dimensions. This would make olfaction not unlike vision and audition, where visual and auditory scene analyses effectively combine space and time information to disambiguate the external world.

## Materials and Methods

### PLIF data

We define the flow direction in the laboratory flume as the x-axis and the lateral direction transverse to the flow as the y-axis. The dye (flourescein) source was located at x = 0 m, y = 0 m and dye was dispersed by turbulent water flow with the mean velocity of 4.6 cm s^−1^. Laser light was emitted by an argon-ion laser at an output intensity of 100mW, which illuminated a vertical light sheet through the water column. When passed through the laser light, flourescein dye (peak absorption at 490 nm) emits light at mean wavelength of 515 nm. Videos of the flouresced dye were recorded within a vertical plane area of 18 × 16 cm centered and parallel to the flow using a 480 × 420 pixel resolution digital camera. An *in situ* calibration was performed to convert pixel intensity to concentration. The dye concentration was measured at downstream positions x = 0.5, 1.0, 1.5, 2.0 and 2.5 m from the source and at cross-stream positions y = 0, 0.05, 0.1 m where y = 0 m is the odor plume centerline (Fig 2). Videos consist of 1025 frames where the frame rate is 60 frames s^−1^. Video recordings were performed 10 times at each location. All images were normalized by the source concentration in each run; therefore dye amplitude in each pixel is represented by a percentage of source concentration. To more accurately reconstruct the dynamics a lobster would experience in the plume, we selected an area of 3 by 3 pixels in each image to reflect the dimension of the single annulus of the lobster antennule (1 mm x 1 mm), which is composed of hundreds of somata and cilia [50]. The time series of odor concentrations sampled by a single annulus are extracted by averaging the 9 pixels intensities to find the odor dynamics at this region. Data are included as supplementary material (S1 and S2 Datasets).

### Computational search model

We simulated a searcher with two olfactory sensors in an odor plume parameterized by the PLIF data. The searcher begins each simulation heading up current (heading angle = 180°). The angle of separation between sensors was 60° and the antennule length (i.e. the distance from the body to each sensor) was set to 5 cm to match the morphology of *P. argus*. Step length was also set to 5 cm. We set the maximum observation time at each position as 10 s and computed the number of steps required to find the source in each of 100 Monte Carlo simulations for each initial position. The searcher finds the odor source if its antennule position is within 5 cm of the source.

Because dye amplitude was measured from cross section images of flow at 15 distinct locations in the plume we selected from each image 3 equally spaced locations in the downstream direction for a total of 45 measurements to build a statistical model that could be used to interpolate odor statistics to all locations visited by simulated searchers. Statistical fluid dynamics can be used to predict the theoretical behavior of various statistics of a turbulent odor plume (e.g., [14]). These methods yield functional forms for the relationship between odor statistics (e.g., mean concentration, the times between odor encounters, the time intervals for which a given threshold is exceeded, etc) and position relative to the plume source. However, because we had access to data from a turbulent plume specifically designed to mimic those experienced by searching marine organisms, we chose to fit odor statistics to data using functional forms that best described observed relationships rather than fitting the forms predicted from theory. For the length scales concerned here, this choice has little bearing on our results and, in fact, the general conclusions we reach about the utility of the time intervals between odor arrivals are consistent with the results of theory.

The time since the last odor threshold up-crossing was well-fitted by an exponential distribution. The parameter for the exponential distribution, i.e., the mean time since the last up-crossing, increased roughly exponentially as the distance from the source increases along the plume centerline [51]:

$$\bar{\Delta }\left(x,0\right)=\Delta_{0}e^{\lambda x},\;x>0,y=0.$$ (2)

In the direction of the cross stream, the mean was assumed to increase exponentially with increasing distance from the plume centerline at a fixed downstream distance as

$$\bar{\Delta }\left(x,y\right)=a\left(x\right)e^{\eta (x)|y|},\;x>0,\left|y\right|=0.$$ (3)

where *a*(*x*) = *g* exp(*hx*) + *n*, *η*(*x*) = *px* ^*q*^ + *n*, and again, the functional forms were chosen based on PLIF data. The parameters *g*, *h*, *p*, and *q* were obtained by fitting the mean values at 45 measured locations. The additive Gaussian noise *n* is zero mean with a standard deviation set equal to the fitting error.

Dye intensity in each of the 15 frame locations was well-fitted by a Gamma distribution and we used this distribution to model instantaneous odor concentration. The mean concentration along the plume centerline was modeled as

$$\bar{C}\left(x,0\right)=C_{0}e^{-\beta x},\;x>0,y=0,$$ (4)

whereas the mean in the cross-stream direction was modeled with a Gaussian function [51, 52],

$$\bar{C}\left(x,y\right)=c\left(x\right)e^{-\frac{y^{2}}{\sigma {\left(x\right)}^{2}}},\;x>0,\left|y\right|>0,$$ (5)

where *c*(*x*) = *k* exp(*bx*) + *n* and *σ*(*x*) = *mx* ^*d*^ + *n*. The parameters *k*, *b*, *m*, and *d* and the noise *n* was again obtained by fitting mean concentration at 45 measurement locations. The mean of the Gamma distribution is the product of its two parameters, i.e., the shape and scale parameters, so we modeled the scale parameter in the same manner as the mean concentration, and compute the shape parameter by dividing mean concentration by the scale parameter.

## Supporting Information

S1 Appendix Supporting information discussing time delay embedding and alternative search strategies. (PDF)

S1 Dataset Dye intensity data from planar laser-induced fluorescence (PLIF) experiments in flume. (MAT)

S2 Dataset Metadata for S1 Dataset describing the contents and structure of the dataset in detail. (RTF)
