# Correction: Neurally Encoding Time for Olfactory Navigation

**DOI:** 10.1371/journal.pcbi.1004742

**Published:** 2016-01-27

**Authors:** The PLOS Computational Biology Staff

An error was introduced during the typesetting process. There are two typographical errors in the x-axis label in Fig 2. The publisher apologizes for these errors.

Please view the correct version of Fig 2 here:

**Fig 2:**
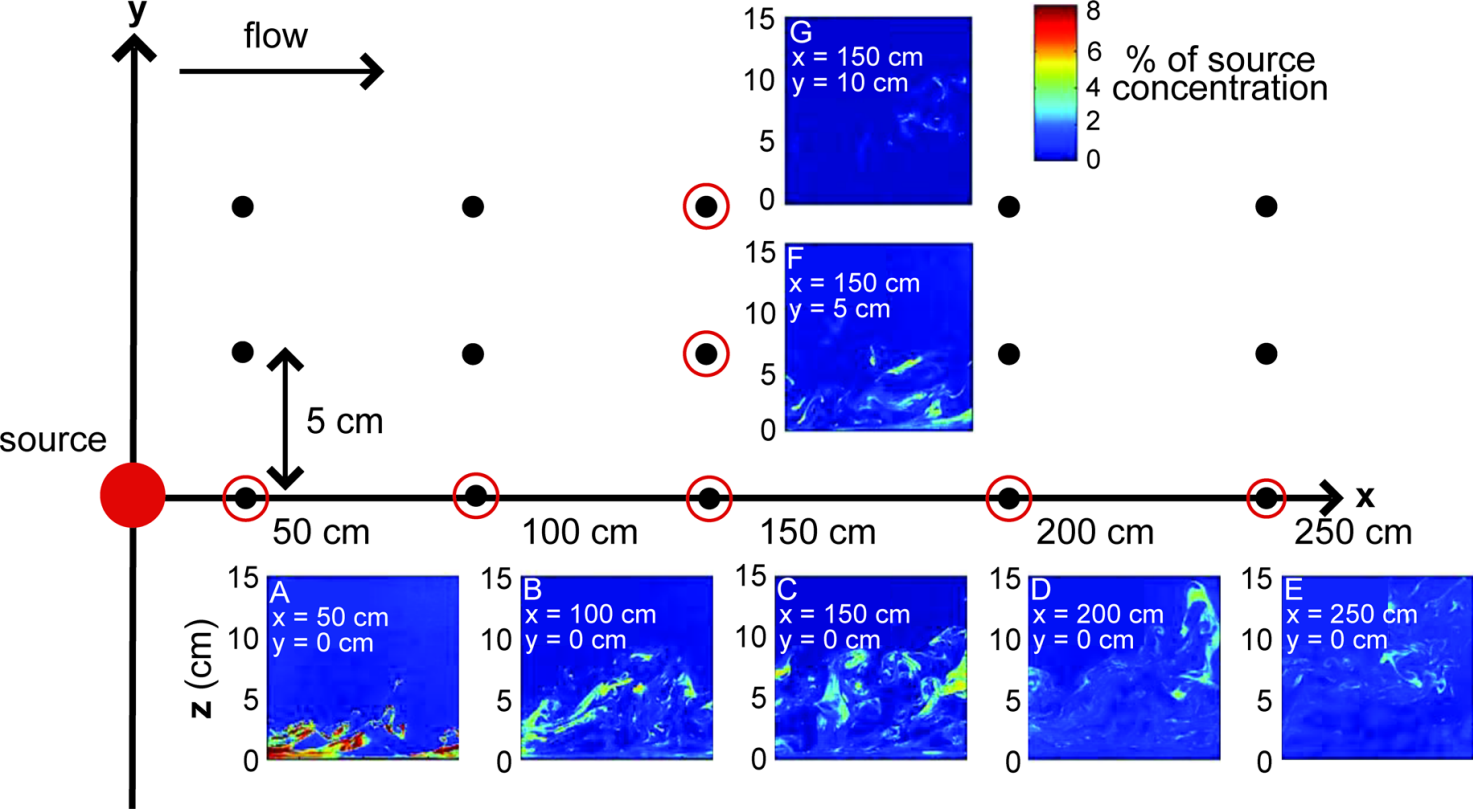
Odor plume PLIF videos taken at 15 locations. Instantaneous odor concentration (expressed as % of source concentration) at (A) x = 50 cm, (B) x = 100 cm, (C) x = 150 cm, (D) x = 200 cm, (E) x = 250 cm from the source along the odor plume centerline, and (F) y = 5 cm, (G) y = 10 cm from the odor plume centerline at x = 150 cm.
