# Supplementary material for: Neurally Encoding Time for Olfactory Navigation
Source: PLoS Comput Biol. 2016 Jan 5;12(1):e1004682. doi: 10.1371/journal.pcbi.1004682 (PMC4711578; doi:10.1371/journal.pcbi.1004682)
Supplement: S1 Appendix — (PDF) [file pcbi.1004682.s001.pdf]

S1 Appendix: Supporting Information for "Neurally Encoding Time for Olfactory Navigation"

by In Jun Park, Andrew M. Hein, Yuriy V. Bobkov, Matthew A. Reidenbach, Barry W. Ache, and Jose C. Principe

Reconstruction of attractor

In order to construct a d dimensional attractor from the time series of odor concentration $\{x(i), i = 1,2,...,N\}$, we build the vectors $\mathbf{x_i} = [x(i), x(i+T), x(i+2T),..., x(i+T(d-1))]$ where T is the delay time and d is the embedding dimension. The sequence $\{\mathbf{x_i}, i = 1,2,..., N-T(d+1)\}$ constitutes a trajectory in the attractor. The delay time T is determined as a small multiple (in this case, 2) of the correlation time that is defined by the time shift where the autocorrelation function of the time series $x(i)$ decays to 1/e [33]. The delay time reflects the idea that consecutive $x(i)$ should be close enough to be dynamically related but far enough to be nearly uncorrelated [33]. The autocorrelation function of the concentration time series at different locations and the obtained time delays (black circle) are shown in Fig A. The embedding dimension is determined by using the false nearest neighbors (FNN) method [S1]. This method examines the number of false nearest neighbors as a function of embedding dimension to find appropriate embedding space. If an embedding dimension is too small, points on the attractor appear to be nearest neighbors although they are actually not. By increasing the embedding dimension, these points are no longer nearest neighbors [S1]. The procedure starts by finding the nearest neighbor of $\mathbf{x_i}$ and defining it as $\mathbf{x_i^{NN}}$ [33,S1]. Then, the distance between two vectors in *d* dimensions is computed as

$$D_d(i) = \left\| \mathbf{x_i} - \mathbf{x_i^{NN}} \right\| = \sqrt{\sum_{m=1}^{d} [x(i+(m-1)T) - x^{NN}(i+(m-1)T)]^2} \qquad \text{(S1)}$$

By increasing the dimension such $d+1$, $D_{d+1}(i)$ is also computed, and $\mathbf{x}_\mathbf{i}^{\mathbf{NN}}$ becomes the false nearest neighbor of $\mathbf{x}_\mathbf{i}$ if the ratio between $D_{d+1}(i)$ and $D_d(i)$ is greater than some threshold.

$$\frac{D_{d+1}(i)}{D_d(i)} > R_{th} \tag{S2}$$

In general, the threshold $R_{th} \geq 10$ effectively identifies false neighbors [S]. Here, we set R_{th} to 30 so that the embedding dimension of zero false nearest neighbor does not exceed 20. Fig A shows the proportion of FNN as a function of the embedding dimension. We selected dimension of 10 where the proportion of false nearest neighbors is less than 1% for all given locations.

Selecting the threshold for recurrence plot construction. The spherical radius, r, for constructing the recurrence plot has to be carefully selected because if it is too small, few trajectories will return to the point $\mathbf{x}_\mathbf{i}$ during the data window; if the radius is too large, all points become neighbors of each other. Following [S2], we chose a value of r located within the linear scaling region of the log-log plot of recurrence rate versus radius corresponding to a recurrence rate of 2% of the maximum recurrence rate, where recurrence rate (RR) is defined as [S3]

$$RR = \sum_{i,j=1}^{N} R(i,j) / N^2 \tag{S3}$$

and should be small (0.1~2 %). Fig. B shows RR as a function of radius for four locations. By setting RR to 2%, the radius falls into the linear scaling region for all given locations.

Search strategies based on a single sensor

In addition to the two-sensor strategy described in the Main Text, we also implemented a search strategy that uses a single sensor for comparison. The strategy using one sensor is based on the stochastic gradients of $\widehat{T_1}$, $\widehat{T_2}$ for both time since the last encounter and

concentration. Given that shorter periods of intermittency are more likely to occur when approaching the source, the searcher attempts to move in the direction of the minimum time since last encounter. Therefore, the strategy is modeled as a gradient descent algorithm. On the other hand, instantaneous concentration tends to increase when approaching the source, therefore the search uses a gradient ascent algorithm. In both strategies, the searcher moves from one position to another and obtains a measurement during a certain period of time. Then, it decides where to move and repeats the cycle. However, if no measurement is acquired within a maximum observation time, the searcher backtracks to its most recent position so that it can stay inside the plume. The gradient is normalized and the searcher moves with a fixed step length to avoid being stuck at one location or deviating from the odor plume. The searching trajectory for the strategy based on the time since last encounter using a single sensor is shown Fig. C. The details of the strategies are described in Table S1. For the strategy using one sensor, we set the step length at 5 cm that is similar to the lobster's antennule size [S4], and the maximum observation time to 10 s. We set the initial heading angle to 120° which is defined as the angle between heading direction and flow direction. The searcher fails if the number of steps exceeds 10^5. Strategies employing two sensors are described in the Main Text.

References

S1. Kennel MB, Brown R, & Abarbanel HDI (1992) Determining embedding dimension for phase-space reconstruction using a geometrical construction. *Phys Rev A* 45(6):3403-3411.

S2. Webber CL & Zbilut JP (2005) Recurrence quantification analysis of nonlinear dynamical systems. *Tutorials in Contemporary Nonlinear Methods for the Behavioural Sciences*, eds Riley MA & Van Orden GC (National Science Foundation, Arlington), pp 26–95.

S3. Marwan N, Romano MC, Thiel M, & Kurths J (2007) Recurrence plots for the analysis of complex systems. *Phys Rep* 438(5–6):237-329.

S4. Goldman JA & Patek SN (2002) Two sniffing strategies in palinurid lobsters. *J Exp Biol* 205(Pt 24):3891-3902

Supporting Figures

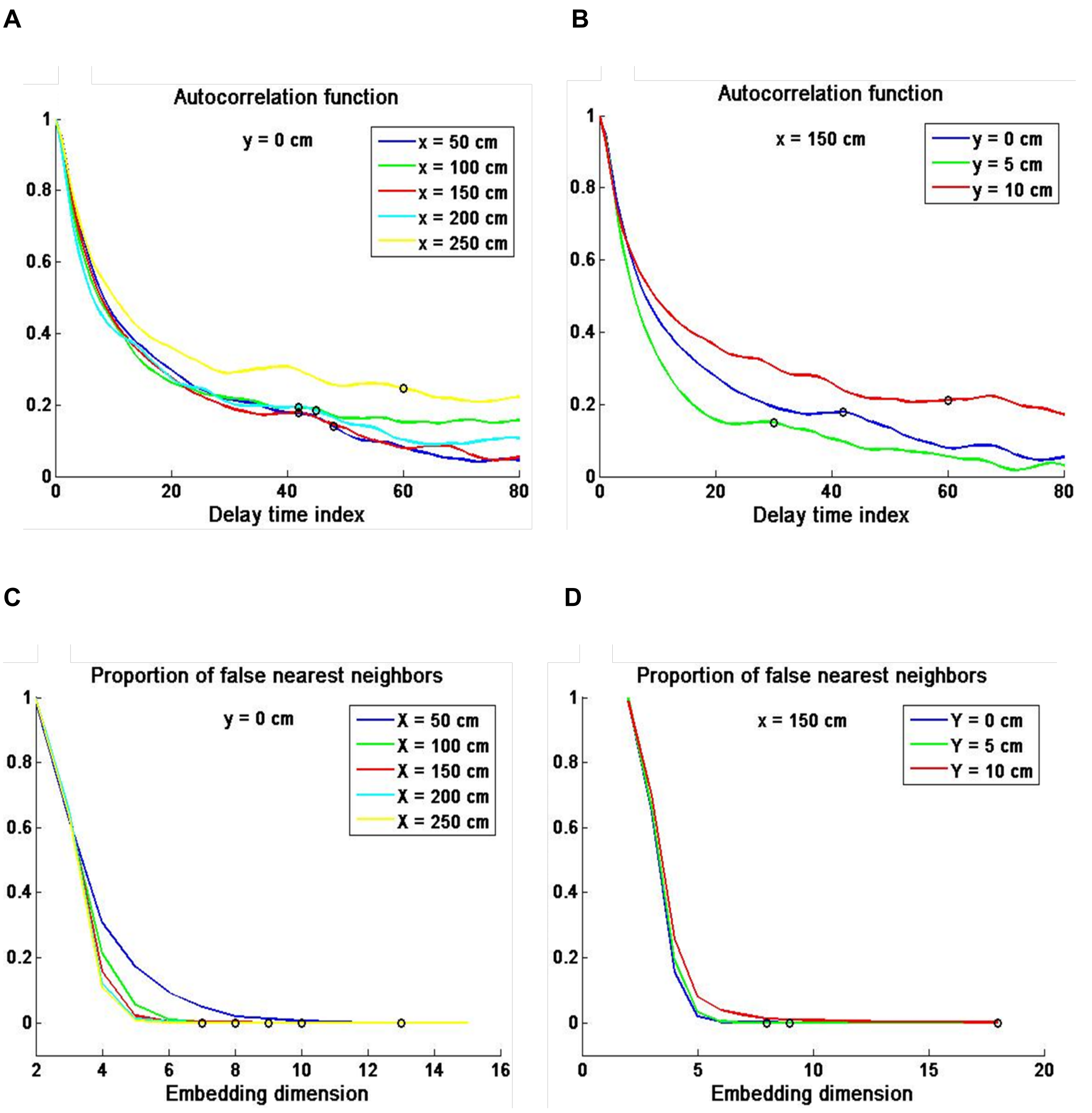

Fig A. Autocorrelation function and the proportion of false nearest neighbors as a function of embedding dimensions. (A) The autocorrelation functions for x = 50, 100, 150, 200, and 250 cm from the source along the plume centerline (obtained time delays: 48, 42, 42, 45, 60, respectively), (B) y = 0, 5, and 10 cm from the centerline at x = 150 cm (obtained time delays: 42, 30, 60, respectively). (C) A proportion of false nearest neighbors as a function of the embedding dimension for x = 50, 100, 150, 200, and 250 cm from the source along the plume

centerline (obtained dimensions: 13, 10, 9, 8, 7, respectively), (D) y=0, 5, 10 cm from the centerline at x = 150 cm (obtained dimensions: 9, 8, 18, respectively).

A **B**

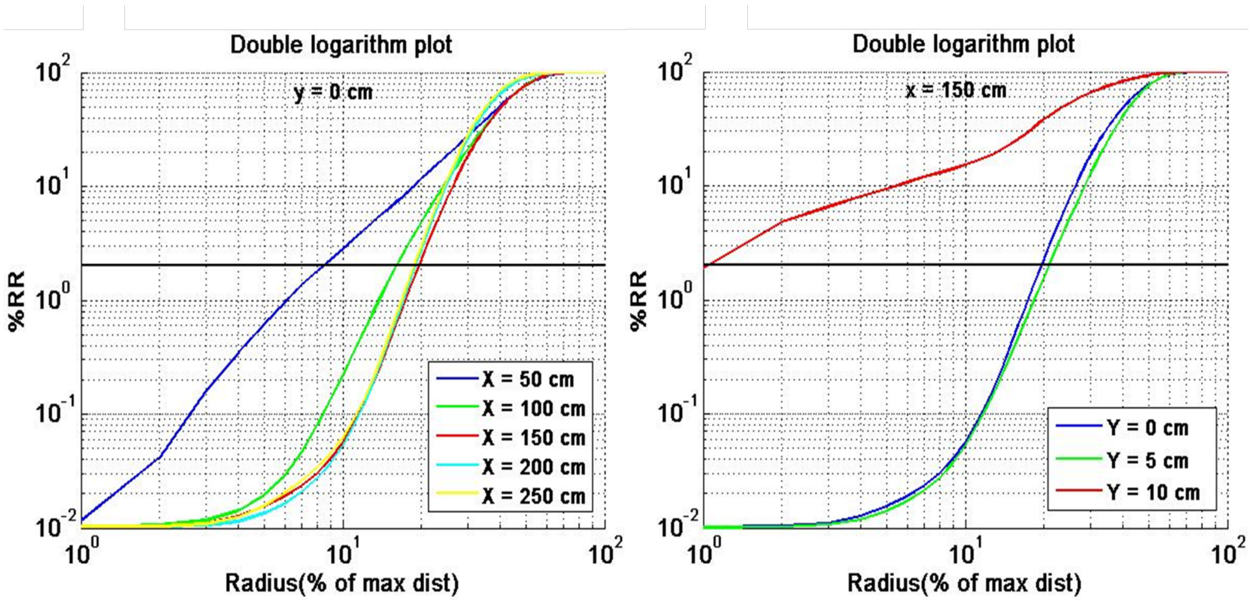

Fig B. Log-log plot of recurrence rate (RR) versus radius for selecting an appropriate radius. Black line represents 2% RR. (A) The radius for 2% RR for x = 50, 100, 150, 200 and 250 cm from the source along the centerline are 8, 16, 20, 19, and 19 % of the maximum distance between attractors, respectively. (B) The radius for 2% RR for y = 0, 5, 10 cm from the centerline at x = 150 cm are 1, 20, and 21 % of the maximum distance between attractors, respectively.

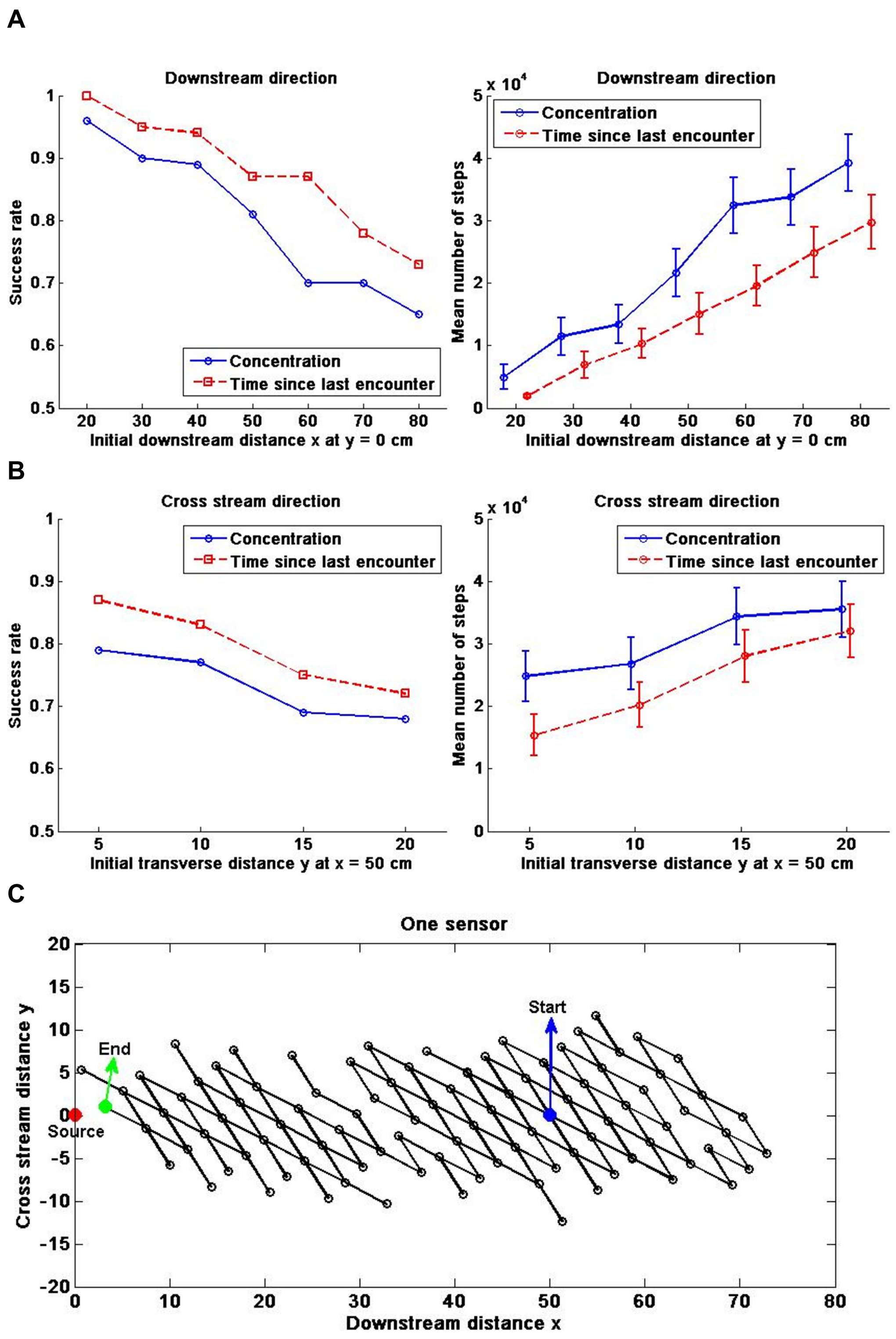
A
Downstream direction
Success rate
Initial downstream distance x at y = 0 cm
Concentration
Time since last encounter
Downstream direction
x 10^4
Mean number of steps
Initial downstream distance at y = 0 cm
Concentration
Time since last encounter
B
Cross stream direction
Success rate
Initial transverse distance y at x = 50 cm
Concentration
Time since last encounter
Cross stream direction
x 10^4
Mean number of steps
Initial transverse distance y at x = 50 cm
Concentration
Time since last encounter
C
One sensor
Cross stream distance y
Downstream distance x
Start
End
Source

Fig C. Performance of single sensor strategies. (A) Success rate (left panel) and mean and standard error for number of steps (right panel) required to reach the source for each strategy using one sensor for the downstream direction, and (B) cross-stream direction. (C) Trajectory of the strategy using one sensor when the time since the last encounter is used for an initial searcher position of x = 50 cm, y = 0 cm. The number of steps to reach the source is 1255.

Tables

Table A. Search strategy based on one sensor

Strategy based on one sensor	
Source location: $\mathbf{s} = [0,0]$.<br>Step length: l<br>Heading angle: θ<br><br>At initial position $\mathbf{r}(1) = [\mathrm{x}(1), \mathrm{y}(1)]$, measure time since last odor encounter $\Delta(1)$ and instantaneous concentration $C(1)$ during observation time $\mathrm{T_{ob}}$.<br><br>Then, random move to<br>$\mathbf{r}(2) = [\mathrm{x}(1) - l \cdot \cos(\theta), \mathrm{y}(1) - l \cdot \sin(\theta)]$.<br><br>Increase the number of steps<br>$\mathrm{n_{step}}(1) = 1$.	
Algorithm 1: Time since last odor encounter	Algorithm 2: Concentration
Repeat until $\|\mathbf{r} - \mathbf{s}\| \leq l$ or $\mathrm{n_{step}} > 10^5$	Repeat until $\|\mathbf{r} - \mathbf{s}\| \leq l$ or $\mathrm{n_{step}} > 10^5$
If $\Delta(\mathrm{i}) \leq \mathrm{T_{ob}}$	*If* $\Delta(\mathrm{i}) \leq \mathrm{T_{ob}}$
compute the gradient:<br>$\mathbf{g}(\mathrm{i}) = \left[\frac{\Delta(\mathrm{i}) - \Delta(\mathrm{i}-1)}{\mathrm{x}(\mathrm{i}) - \mathrm{x}(\mathrm{i}-1)}, \frac{\Delta(\mathrm{i}) - \Delta(\mathrm{i}-1)}{\mathrm{y}(\mathrm{i}) - \mathrm{y}(\mathrm{i}-1)}\right]$	compute the gradient:<br>$\mathbf{g}(\mathrm{i}) = \left[\frac{\mathrm{C}(\mathrm{i}) - \mathrm{C}(\mathrm{i}-1)}{\mathrm{x}(\mathrm{i}) - \mathrm{x}(\mathrm{i}-1)}, \frac{\mathrm{C}(\mathrm{i}) - \mathrm{C}(\mathrm{i}-1)}{\mathrm{y}(\mathrm{i}) - \mathrm{y}(\mathrm{i}-1)}\right]$
normalize the gradient:<br>$\hat{\mathbf{g}}(\mathrm{i}) = \mathbf{g}(\mathrm{i}) / \|\mathbf{g}(\mathrm{i})\|$	normalize the gradient:<br>$\hat{\mathbf{g}}(\mathrm{i}) = \mathbf{g}(\mathrm{i}) / \|\mathbf{g}(\mathrm{i})\|$
move to new location:<br>$\mathbf{r}(\mathrm{i}+1) = \mathbf{r}(\mathrm{i}) - l \cdot \hat{\mathbf{g}}(\mathrm{i})$	move to new location:<br>$\mathbf{r}(\mathrm{i}+1) = \mathbf{r}(\mathrm{i}) + l \cdot \hat{\mathbf{g}}(\mathrm{i})$
increase the number of steps:<br>$\mathrm{n_{step}}(i) = \mathrm{n_{step}}(i-1) + 1$.	increase the number of steps:<br>$\mathrm{n_{step}}(i) = \mathrm{n_{step}}(i-1) + 1$.
else	*else*
move to the previous position:<br>$\mathbf{r}(\mathrm{i}+1) = \mathbf{r}(\mathrm{i}-1)$.	move to the previous position:<br>$\mathbf{r}(\mathrm{i}+1) = \mathbf{r}(\mathrm{i}-1)$.

increase the number of steps: $n_{step}(i) = n_{step}(i-1) + 1$.	increase the number of steps: $n_{step}(i) = n_{step}(i-1) + 1$.

Table B. Search strategy based on two sensors

Strategy based on two sensors

Source location: $\mathbf{s} = [0,0]$.
Step length: l
Antennule length: l
Heading angle: θ

At initial position $\mathbf{r}(1) = [x(1), y(1)]$, measure $\Delta_L(1)$ at $\mathbf{r_L}(1) = \mathbf{r}(1) + l \cdot [\cos(\theta - 30^o), \sin(\theta - 30^o)]$ and $C_L(1)$ using left antennule during observation time T_{ob}. Likewise, $\Delta_R(1)$ at $\mathbf{r_R}(1) = \mathbf{r}(1) + l \cdot [\cos(\theta + 30^o), \sin(\theta + 30^o)]$ and $C_R(1)$ using right antennule during observation time T_{ob}.

The number of steps is
$n_{step}(1) = 0$.

Algorithm 3: Time since last odor encounter	Algorithm 4: Concentration
Repeat until $\lVert\mathbf{r_L} - \mathbf{s}\rVert \leq l$ or $\lVert\mathbf{r_R} - \mathbf{s}\rVert \leq l$ or $n_{step} > 10^5$	Repeat until $\lVert\mathbf{r_L} - \mathbf{s}\rVert \leq l$ or $\lVert\mathbf{r_R} - \mathbf{s}\rVert \leq l$ or $n_{step} > 10^5$
If $\Delta_L(i) \leq T_{ob}$ and $\Delta_R(i) \leq T_{ob}$	*If* $\Delta_L(i) \leq T_{ob}$ and $\Delta_R(i) \leq T_{ob}$
if $\Delta_L(i) < \Delta_R(i)$<br>$\mathbf{r}(i+1) = \mathbf{r_L}(i)$<br>$n_{step}(i) = n_{step}(i-1) + 1$<br>else<br>$\mathbf{r}(i+1) = \mathbf{r_R}(i)$<br>$n_{step}(i) = n_{step}(i-1) + 1$	if $C_L(i) > C_R(i)$<br>$\mathbf{r}(i+1) = \mathbf{r_L}(i)$<br>$n_{step}(i) = n_{step}(i-1) + 1$<br>else<br>$\mathbf{r}(i+1) = \mathbf{r_R}(i)$<br>$n_{step}(i) = n_{step}(i-1) + 1$
else if $\Delta_L(i) \leq T_{ob}$ and $\Delta_R(i) > T_{ob}$<br>$\mathbf{r}(i+1) = \mathbf{r_L}(i)$<br>$n_{step}(i) = n_{step}(i-1) + 1$	*else if* $\Delta_L(i) \leq T_{ob}$ and $\Delta_R(i) > T_{ob}$<br>$\mathbf{r}(i+1) = \mathbf{r_L}(i)$<br>$n_{step}(i) = n_{step}(i-1) + 1$
else if $\Delta_L(i) > T_{ob}$ and $\Delta_R(i) \leq T_{ob}$<br>$\mathbf{r}(i+1) = \mathbf{r_R}(i)$<br>$n_{step}(i) = n_{step}(i-1) + 1$<br>*else*<br>Turn back and move one step forward:<br>$\theta = \theta + 180^o$	*else if* $\Delta_L(i) > T_{ob}$ and $\Delta_R(i) \leq T_{ob}$<br>$\mathbf{r}(i+1) = \mathbf{r_R}(i)$<br>$n_{step}(i) = n_{step}(i-1) + 1$<br>*else*<br>Turn back and move one step forward:<br>$\theta = \theta + 180^o$

$\mathbf{r}(\mathrm{i}+1)=\mathbf{r}(\mathrm{i})+l\cdot[\cos(\theta),\sin(\theta)]$<br>$\mathrm{n}_{\mathrm{step}}(i)=\mathrm{n}_{\mathrm{step}}(i-1)+1$	$\mathbf{r}(\mathrm{i}+1)=\mathbf{r}(\mathrm{i})+l\cdot[\cos(\theta),\sin(\theta)]$<br>$\mathrm{n}_{\mathrm{step}}(i)=\mathrm{n}_{\mathrm{step}}(i-1)+1$
