# Supplementary material for: Neurally Encoding Time for Olfactory Navigation
Source: PLoS Comput Biol. 2016 Jan 5;12(1):e1004682. doi: 10.1371/journal.pcbi.1004682 (PMC4711578; doi:10.1371/journal.pcbi.1004682)
Supplement: S2 Dataset — (RTF) [file pcbi.1004682.s003.rtf]

S2 Dataset: Description of contents of S1 Dataset from “Neurally Encoding Time for Olfactory Navigation” by Park et al.Dataset contains dye intensity data from planar laser-induced fluorescence (PLIF) experiments in flume. Each cell in array contains data from one of the 45 locations at which PLIF data were collected. Columns in cell array represent distance from the source in the x-direction (51, 59, 67, 101, 109, 117, 151, 159, 167, ,201, 209, 217, 251, 259, and 267 cm from source); rows represent distance in the y-direction, transverse to the plume major axis (0, 5, and 10 cm). For each location, the dye intensity in each frame of the 1025 video frames is reported. The 10 replicate sequences of 1025 frames each are concatenated. 
